# Vertebral Adaptations to Large Body Size in Theropod Dinosaurs

**DOI:** 10.1371/journal.pone.0158962

**Published:** 2016-07-21

**Authors:** John P. Wilson, D. Cary Woodruff, Jacob D. Gardner, Holley M. Flora, John R. Horner, Chris L. Organ

**Affiliations:** 1 Department of Earth Sciences, Montana State University, Bozeman, MT, 59717, United States of America; 2 Department of Paleontology, Museum of the Rockies, Bozeman, MT, 59717, United States of America; 3 Department of Microbiology and Immunology, Montana State University, Bozeman, MT, 59717, United States of America; University of Naples, ITALY

## Abstract

Rugose projections on the anterior and posterior aspects of vertebral neural spines appear throughout Amniota and result from the mineralization of the supraspinous and interspinous ligaments via metaplasia, the process of permanent tissue-type transformation. In mammals, this metaplasia is generally pathological or stress induced, but is a normal part of development in some clades of birds. Such structures, though phylogenetically sporadic, appear throughout the fossil record of non-avian theropod dinosaurs, yet their physiological and adaptive significance has remained unexamined. Here we show novel histologic and phylogenetic evidence that neural spine projections were a physiological response to biomechanical stress in large-bodied theropod species. Metaplastic projections also appear to vary between immature and mature individuals of the same species, with immature animals either lacking them or exhibiting smaller projections, supporting the hypothesis that these structures develop through ontogeny as a result of increasing bending stress subjected to the spinal column. Metaplastic mineralization of spinal ligaments would likely affect the flexibility of the spinal column, increasing passive support for body weight. A stiff spinal column would also provide biomechanical support for the primary hip flexors and, therefore, may have played a role in locomotor efficiency and mobility in large-bodied species. This new association of interspinal ligament metaplasia in Theropoda with large body size contributes additional insight to our understanding of the diverse biomechanical coping mechanisms developed throughout Dinosauria, and stresses the significance of phylogenetic methods when testing for biological trends, evolutionary or not.

## Introduction

**Fig 1:**
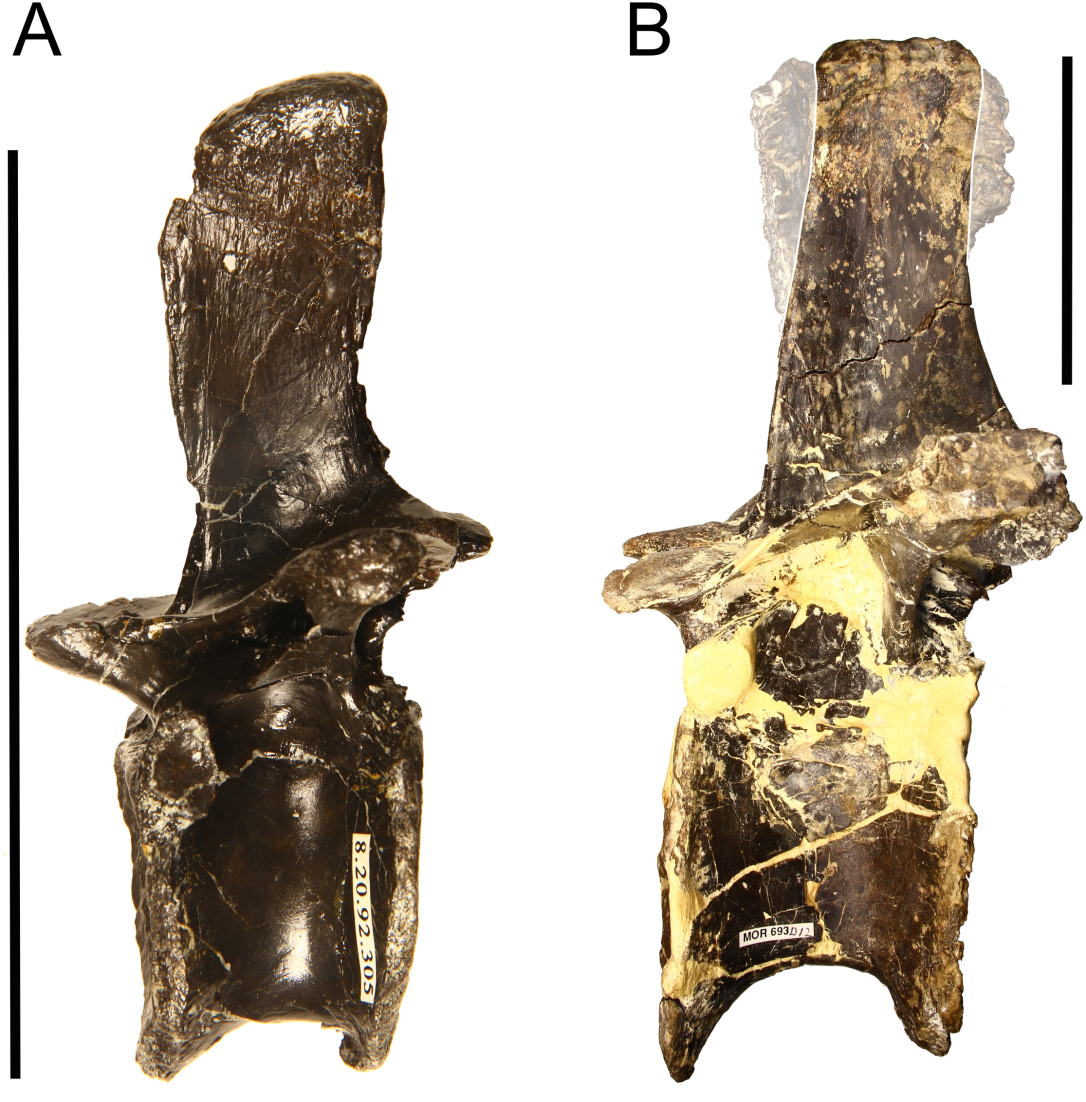
Dorsal vertebrae showing evidence of metaplastic rugosities on the neural spine. (A) Dorsal vertebra from a *Troodon* (MOR 553–8.20.92.305), a small-bodied theropod. (B) A dorsal vertebra from an Allosaurus (MOR 693), a large bodied theropod. Note the expanded metaplastic rugosities in *Allosaurus* (highlighted by 50% transparency) compared with *Troodon*. of the mature individual compared with the smaller rugosities of the immature animal. Both dorsal vertebrae in left lateral view. Scale bars = 10 cm.

Many large-bodied amniotes, from bovids to crocodilians, exhibit rugose, spur-like projections on the neural spines within the dorsal and caudal regions of the spinal column [1]. These projections are thought to arise from metaplasia of the supraspinous and interspinous ligaments [1–4] caused by tensile loading in the apical portion of the neural spines, as would be predicted from ventrally directed bending stresses of the vertebral column [5]. Metaplasia, in the paleontological and evolutionary biological sense, is the permanent transformation of tissue types, and cannot be described as “ossification” of the ligaments, as metaplasia is not the process of bone formation [4]. Anteroposterior and apical neural spine projections, although widespread taxonomically, nevertheless lack uniform distribution within the vertebral column and vary widely from individual to individual within extant species. In mammals this condition is often a stress-induced pathology and described as “spondylitis ossificans ligamentosa”, “spondylosis hyperostotica”, “physiologic vertebral ligament calcification”, or “ankylosing hyperostosis of the spine” [6–8]. In birds the metaplasia of vertebral ligaments is common, especially in species that also possess “ossified” tendons [9]. In the fossil record of non-avian theropod dinosaurs, these structures appear to be common in large-bodied taxa (Fig 1B). This is not surprising, as stress-induced bone remodeling and other physiological responses to stress are well-documented within amniotes, including dinosaurs [10]. The vertebral column provides biomechanical support for the trunk and tail, an especially important functional role given the bipedal posture of nearly all non-avian theropods [11]. For these species, a large body mass would subject the spinal column to great tensile stress, especially in the anterior/posterior edges and apex of the neural spines in the dorsal, sacral, and caudal regions. Moreover, bending moments along the posterior section of the dorsal region and the anterior section of the caudal region would increase during locomotion as the hip extensors/flexors generated forces throughout the gait cycle. A stiff spinal column would facilitate force transfer to the limbs rather than deforming the vertebral column during contraction of the hip flexors. The potential function of the spinal metaplastic tissue is therefore hypothesized to provide passive support for body weight by extending hard tissue, supporting the epaxial muscles, and increasing locomotor efficiency through increased spinal rigidity.

Bone surface rugosities are thought to be produced by frequent application of stress during development [12]. According to Wolff’s law, the principle direction of bone remodeling occurs along lines of stress [13,14]. Metaplastic spinal projections are oriented in the cranial-caudal direction, suggesting that tensile loading of the interspinous and supraspinous ligaments metaplastically transformed these tissues. This implies that the projections were a physiological, not an evolutionary (genetic), adaptation. It is common for the presence of these metaplastic spinal structures to be noted in specimen descriptions, often as “interspinal ligament scars,” though until now they have neither been histologically examined nor evaluated in a comparative phylogenetic context, though this feature has been used in phylogenetic inference to reconstruct evolutionary relationships (e.g. [15,16]). Here we test the hypothesis that mean body size (using femur length as a proxy), is different between species that exhibit metaplastic projections on the dorsal neural spines and species that lack them. We also consider evidence suggesting that the projections enlarge during ontogeny.

## Materials and Methods

Institutional Abbreviations for this study are as follows: BMRP—Burpee Museum of Natural History, Rockford, IL, USA; FMNH—Field Museum of Natural History, Chicago, IL, USA; MOR—Museum of the Rockies, Bozeman, MT, USA; TMP—Royal Tyrrell Museum of Paleontology, Drumheller, Alberta, CA; USNM—National Museum of Natural History, Washington, DC, USA. Neural spines from *Tyrannosaurus rex* (MOR 555, MOR histological project number 2002–5), domestic turkey *Meleagris gallopavo* (MOR histological project number 2004-09R), and *Alligator mississippiensis* (MOR histological project number 2004-08R) were sampled for histological analysis. Transverse and longitudinal sections were sampled from *Meleagris* between the neural spine of the notarium and the free dorsal vertebra. Transverse and longitudinal sections were sampled from the neural spine of the last two thoracic vertebrae of *Alligator*. The neural spine of the *Tyrannosaurus* was likewise sectioned transversely and longitudinally. The *Meleagris* and *Alligator* samples were fixed in 10% neutral buffered formalin for storage and stained with hematoxylin and eosin (H&E) and trichrome to note the occurrence of collagen. We followed standard paleohistological techniques to prepare thin sections [17]. Finished slides were photographed using a Nikon Optiphot-Pol polarizing microscope equipped with a Nikon DS-Fi1 digital camera, and compiled with NIS-Elements BR 3.0 software. Microanatomical terminology used here follows established nomenclature for bone and metaplastic tissue [2,4,18–20].

Metaplastic projections along the anterior and posterior edges and apex of the neural spines in the dorsal region of the vertebral column of non-avian theropods were coded as absent (0) or present (1). There are theropod taxa in which metaplastic spinal projections occur solely in the caudal vertebrae and not in the dorsal neural spines. These were coded as a 0, as projections solely on caudal neural spines would provide little weight-bearing support to the spinal column anterior to the hind limbs, and most likely only provided increased locomotion efficiency. Spinal apex expansion, which appears as a rounded, lateral flaring of the spine at its apex is not due to the same physiological or biological processes as the metaplasia of the interspinal ligaments examined here and therefore coded as a 0. Vertebrae exhibiting metaplasia of both the supraspinous and interspinous ligaments, in which the projections span the entire height and apex of the spine, were coded as a 1. Spines on which the metaplastic tissue does not necessarily span the height of the spine but does project beyond the anterior and posterior margins of the spine, so as to contribute to the anteroposterior length of the spine, were also coded as a 1. Ligament metaplasia and scarring that is visible but does not project outwards from the margins of the neural spines, such as in *Troodon*, is coded as a 0, as is complete absence of scarring and projecting tissue. There are some taxa, such as *Compsognathus*, *Dilophosaurus*, *Ornitholestes*, *Huaxiagnathus*, *Scipionyx*, and *Sinosauropteryx*, that possess “hook-like ligament” attachments” on their dorsal neural spines [21]. In *Dilophosaurus*, these are described as “shoulders” that project from the anterior and posterior faces of the neural spines [22]. These features are are periosteal rather than metaplastic. For example, in the coding scheme of Loewen et al., 2013, *Compsognathus*, *Dilophosaurus*, *Ornitholestes*, and *Sinosauropteryx* are all coded as “0 –absent or weakly developed” for character 335 “Dorsal vertebrae, rugose ligament attachment scars on anterior and posterior surfaces of neural spine.”

Therefore, these taxa lack interspinal ligament metaplasia and are coded as 0. We used femur length as a proxy for body size/mass, which has been demonstrated to be a reliable indicator of body mass for bipedal non-avian theropods [23]. Femur length and spinal projection presence/absence are detailed in Table 1. Data from 56 theropod species were collected.

**Table 1:** Femur length and the presence or absence of rugose projections that extend from the anterior and posterior blades of the neural spines in the dorsal region.

Taxon	Femur Length (cm)	Metaplastic Projections (present 1, absent 0)	Femur References	Rugosity References
*Achillobator*	50.5	0	[49]	[50]
*Acrocanthosaurus*	127.7	1	[51]	[52]
*Albertosaurus*	106.6	1	[51]	TMP-81.10.1
*Allosaurus*	100.1	1	[51]	MOR 693
*Archaeopteryx*	5.8	0	[53]	[54]
*Archaeornithomimus*	31.4	0	[55]	[55]
*Avimimus*	18.6	0	[56]	[57]
*Bambiraptor*	17	0	[58]	[58]
*Baryonyx*	120*	1	[59]	[59]
*Buitreraptor*	14.5*	0	[60]	[60]
*Carnotaurus*	103	1	[61]	[61]
*Caudipteryx*	15.2	0	[62]	[63]
*Ceratosaurus*	62	0	[51]	[64]
*Chirostenotes*	31	0	[65]	[66]
*Citipati*	41.1	0	[67]	[68]
*Coelophysis*	21.74	0	[56]	[69]
*Coelurus*	21	0	[56]	[64]
*Compsognathus*	11	0	[41]	[70]
*Daspletosaurus*	100	1	[51]	MOR 1130
*Deinonychus*	33.6	0	[71]	[71]
*Dilophosaurus*	58.7	0	[72]	[22]
*Eoraptor*	15.2	0	[73]	[73]
*Falcarius*	34	0	[56]	[74]
*Fukuiraptor*	50.7	0	[56]	[75]
*Garudimimus*	49.9	1	[56]	[76]
*Guanlong*	41.6	0	[77]	[78]
*Herrerasaurus*	35.4	0	[56]	[79]
*Huaxiagnathus*	16.3	0	[80]	[80]
*Jinfengopteryx*	7.032	0	[81]	[81]
*Khaan*	18.8	0	[68,82]	[82]
*Liliensternus*	44.9	0	[83]	[83]
*Majungasaurus*	56.80	1	[84]	[85]
*Masiakasaurus*	19.36	0	[86]	[87]
*Mei*	8.1	0	[88]	[88]
*Microraptor*	7.59	0	[89]	[90]
*Microvenator*	12.4	0	[91]	[91]
*Mirischia*	16.5	0	[92]	[92]
*Mononykus*	13.84	0	[93]	[93]
*Neovenator*	75	1	[94]	[94]
*Ornitholestes*	21.00	0	[95,96]	[95]
*Ornithomimus*	50	0	[97]	[98]
*Rahonavis*	8.8	0	[99,100]	[100]
*Saurornithoides*	14	0	[101,102]	[101]
*Saurornitholestes*	22.5	0	[99]	[103]
*Shenzhousaurus*	19.1	0	[104]	[104]
*Shuvuuia*	12.45	0	[56]	[105]
*Sinosauropteryx*	8.64	0	[41,106]	[106]
*Sinovenator*	11.11	0	[107]	[107]
*Spinosaurus*	61	0	[108]	[109]
*Staurikosaurus*	23.5	0	[110,111]	[110]
*Struthiomimus*	51.3	0	[97,112]	[112]
*Torvosaurus*	83	1	[56]	[113]
*Troodon*	30	0	[114,115]	MOR 796
*Tyrannosaurus*	134.25	1	[51]	MOR 555
*Tyrannotitan*	140	1	[116]	[117]
*Velociraptor*	18.7	0	[56]	[118]

* Femur length estimated in original publication.

The phylogenetic tree used to analyze character evolution was extracted from a larger phylogeny of dinosaurs [24]. Comparative phylogenetic analysis of body size and the presence of metaplastic projections was performed to normalize for common descent [25] using the program BayesTraits [26]. We used a regression model where the dependent variable was femur length (ln cm) and the independent variable was binary (0 or 1) corresponding to the presence or absence of metaplastic projections on neural spines; amounting to a phylogenetic t-test [27]. This tests whether there is a significant difference in body size (ln femur length) in theropods with and without metaplastic spinal projections. The parameters λ (phylogenetic signal), δ (early vs late bursts), and κ (degree to which character evolution is independent of branch lengths, a measure of punctuation) were estimated as well [26]. These parameters scale the phylogenetic tree to model deviations from a Brownian motion model.

We used log-Bayes factors and posterior distributions of parameter estimates for hypothesis testing [28]. To test for the potential of homology to explain our data we performed a reversible-jump Markov chain Monte Carlo (MCMC) algorithm to estimate ancestral states [29]. We used an exponential hyperprior for the transition rates seeded randomly from zero to ten. All MCMC analyses ran for 5,000,000 iterations with a 100,000 burn-in and a sampling frequency of 1,000.

## Results

**Fig 2:**
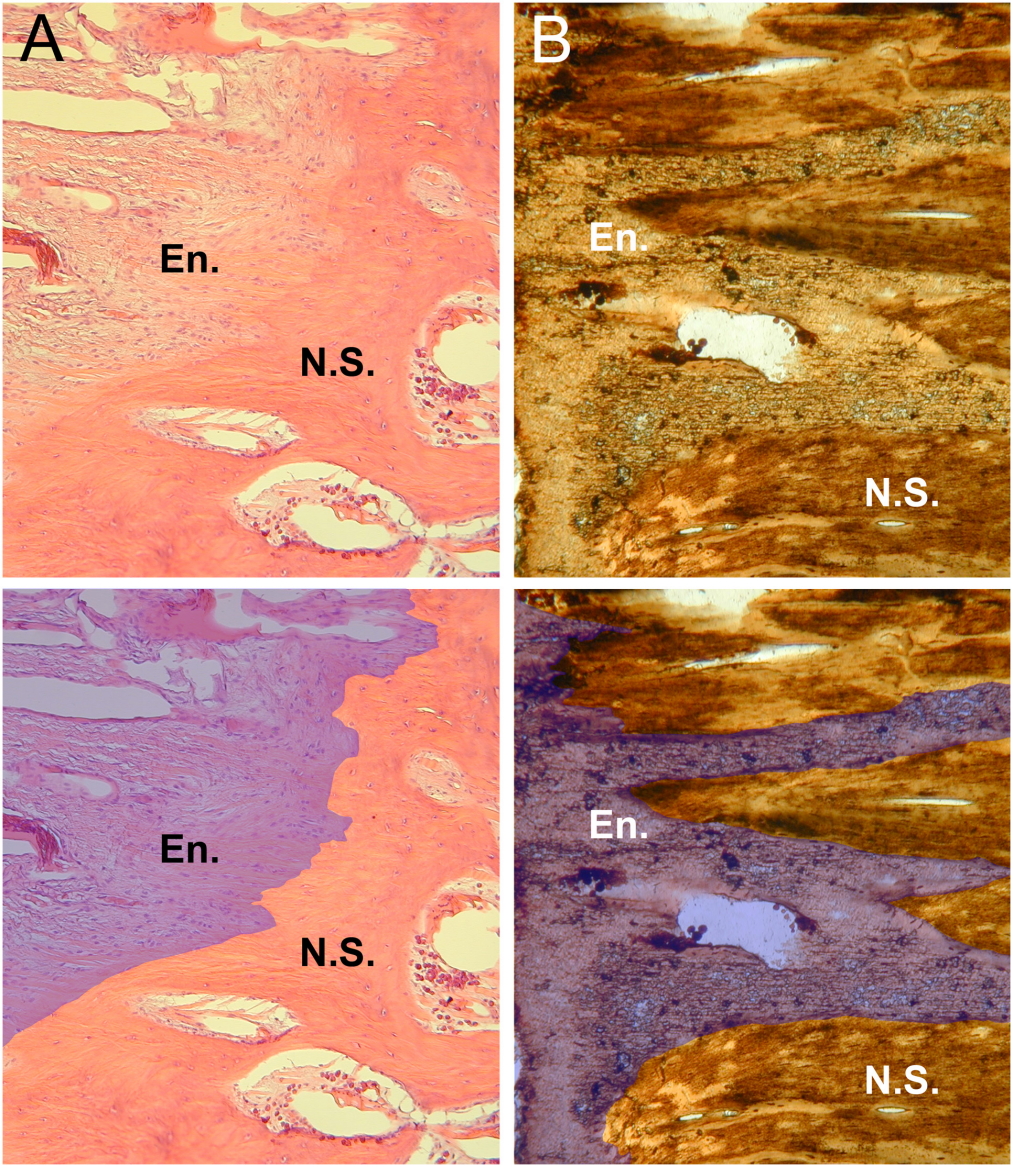
Histological section of the interspinous ligament enthesis in a dorsal vertebra in *Alligator* and *Tyrannosaurus*. (A) The enthesis in *Alligator* (H&E staining, 40x) exhibiting an undulating interface surface between the lighter ligament (marked “En.” on the left of the panel) and bone of the neural spine (marked “N.S.”). (B) The enthesis in *Tyrannosaurus* (100x) shows rough-bundled metaplastic tissue in the area of the enthesis, which deeply interdigitates with the neural spine.

The neural spines of vertebrae within the dorsal region of the *Alligator* and *Meleagris* are capped with a cartilaginous sesamoid. Sesamoids act to protect the ligament from tearing and to increase the ligament’s mechanical effect [30,31]. The sesamoid extends the anteroposterior length of the neural spine where the *ligamentum apicum dorsalis* (supraspinous ligament) attaches. Within the *Alligator* and *Tyrannosaurus* thin sections (Fig 2), there is an irregular, “ribbon”-like tissue that interdigitates into the cortex of the neural spine. This irregular zone in both species is indicative of the metaplastic transitionary tissue known as the enthesis, which is the hypermineralized calcified fibrocartilage from connective tissue (ligament or tendon) to the spine, confirming the identity of the spinal projections as metaplastic [4,32,33]. Similar vertebral entheses have been described from neosauropod neural spines [1,4,32,34]. In the *Tyrannosaurus* sample, this “veneer”-like enthesis pervades into the highly pneumatized neural spine, composed of highly fibrous secondary reconstructions, which agrees with the observation of Horner et al. 2015 [4] showing that the bulk of the neural spine is composed of metaplastic tissue. These data strongly suggest that the dorsal neural spines of *Tyrannosaurus* underwent substantial remodeling during ontogeny, not typically observed in other vertebrates.

**Fig 3:**
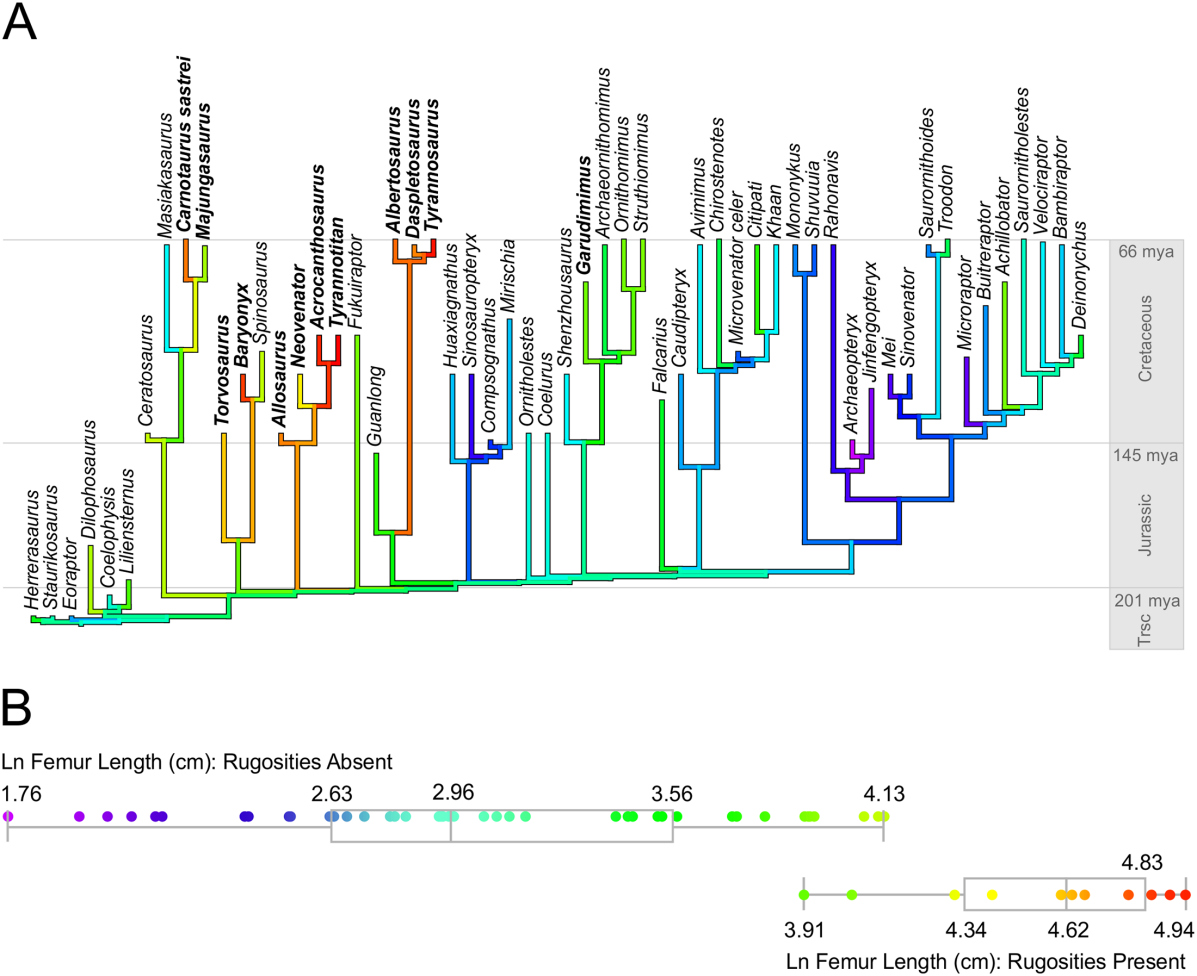
(A) Phylogenetic tree onto which femur length (Ln cm) is mapped using maximum likelihood. Species with metaplastic rugosities on neural spines are bolded. Note that *Spinosaurus* is large-bodied, but lacks rugose neural spines, most likely owing to its elongate neural spines. (B) Distribution of femur length (Ln cm) in theropods grouped by the absence (top quartile plot) or presence (bottom quartile plot) of rugose neural spines. Quantiles are shown in gray boxes with the minimum, 25%, median, 75%, and maximum values shown for each group. The color code of data matches the gradient from the phylogenetic mapping in panel A, which correspond to natural log femur length with red representing the longest and purple representing the shortest. A phylogenetic t-test supports a substantial difference in average body size between species that exhibit or lack neural spine rugosities (n = 56, p-value < 1.0e^-10^).

Our character analysis results are consistent with the hypothesis that metaplastic spinal projections in non-avian theropods were an adaptation to large body size (Fig 3). The model with λ had a harmonic mean (of the log-likelihood) of -63, whereas the model with and λ+δ had a harmonic mean of -49. However, the model with λ+κ had a harmonic mean of -46, and is therefore favored over the λ+δ model by a log Bayes factor of six. This model (phylogenetic t-test) supports a significant difference in body size between the two groups because 100% of the posterior distribution of β_2_ (slope) deviates from the null value of 0 (posterior model parameters: β_1_ = 3.24 (σ = 0.21), β_2_ = 0.88 (σ = 0.23), λ = 0.88 (σ = 0.1), and κ = 0.14 (σ = 0.11). Our RJMCMC ancestral state reconstruction suggests that all nodes along the spine of the phylogeny are associated with the lack of metaplastic projections (all posterior probabilities of character state zero were greater than 95%). This result is in disagreement with the hypothesis that such spinal projections are a homologous feature of theropods and supports our hypothesis that metaplastic projections on neural spines appear numerously throughout the theropod tree as physiological responses to large body size.

## Discussion and Conclusion

The histological data presented here reveals large amounts of metaplastic tissue in the neural spines of *Tyrannosaurus rex*. Together with our phylogenetic analysis, these results suggest that the dorsal aspect of the vertebral column was subject to ventrally directed forces sufficient to induce substantial amounts of metaplasia in the interspinous and supraspinous ligaments of large-bodied non-avian theropods. Stiffening the vertebral column in large-bodied theropods has significant implications for our understanding of their biology. For instance, without support the dorsal, sacral, and caudal regions of the vertebral column would tend to sag or bend ventrally under their own weight. A ventrally bent vertebral column might affect locomotion because hip flexors originate along the ventral aspect of the vertebral column, and rigid support for the biomechanical lever systems would increase the efficiency of locomotion. The interspinous and supraspinous ligaments would help passively resist the tendency of the column to flex or sag ventrally, as would contraction of the epaxial muscles actively. Stiffening these ligaments through metaplasia would improve the spinal column’s ability to resist sagging from body weight or muscle contraction associated with locomotion. Moreover, rigidity in the dorsal region of the vertebral column is thought to assist in theropod breathing [35]. Stiffening the vertebral column through metaplastic mineralization of the interspinous and supraspinous ligaments may have simultaneously facilitated ventilation and limited vertebral mobility in large-bodied theropods. Similar interpretations were recently made by Foth et al [36] regarding the hypothesized function and probable exclusivity of these structures to large bodied theropod taxa, though they describe the preserved ligaments as “ossified,” which they are not.

Theropods in the family Spinosauridae (*Spinosaurus* and kin) are large-bodied, but exhibit both neural spine conditions. *Baryonyx* possesses short neural spines that bear metaplastic projections, while *Spinosaurus* exhibits long neural spines and lacks metaplastic projections. We hypothesize that this absence is due to its elongate neural spines that, like a cantilever bridge, would help distribute stresses along the vertebral column. The elongate neural spines of *Deinocheirus mirificus* [37], which do not bear the spur-like metaplastic projections we discuss here, may have functioned similarly. Other dinosaurian groups also exhibit osteological and metaplastic modifications of the vertebral column that may have functioned analogously to the hypothesized function of the metaplastic projections analyzed here. Numerous ornithischian species exhibit an interwoven network of “ossified” tendons along the lateral aspects of the neural spines in the dorsal and caudal series. This tendon network is associated with *M*. *transversospinalis* [9] and is hypothesized to have stiffened the torso and tail [38]. These tendons, though widely referred to as “ossified,” also seem to have mineralized via the process of metaplasia [4]. Other modifications are found in ornithischians, such as the interlocking zygapophyses and neural spines in the tails of ankylosaurs [4,39] and the three-dimensional myorhabdoi (or “caudal basket”) in Pachycephalosauria [40]. Vertebral modifications also appear in saurischians besides the metaplastic spinal projections of large bodied theropods. Some small bodied dromaeosaurid theropods, like *Deinonychus*, possessed exaggeratedly long caudal vertebral processes and chevrons which have been hypothesized as having mineralized via metaplasia rather than ossification [4], though the function of these modifications most likely differs from that of the spinal projections examined here. As stated in the Methods section, some small-bodied theropod taxa, including *Compsognathus*, *Ornitholestes*, *Huaxiagnathus*, *Scipionyx*, and *Sinosauropteryx*, feature “hook-like ligament attachments” on their dorsal neural spines [21]. Similarly, the moderately-sized *Dilophosaurus*, possesses “shoulders” that project from the anterior and posterior faces of the neural spines [22]. These structures appear to be part of the osseous neural spines themselves and not metaplastic ligaments, as they are structurally dissimilar from the characteristic spur-like projections we discuss. Peyer (2006) states that these hook-like structures, along with fan-shaped anterior neural spines [41], are considered diagnostic of compsognathids. Because these structures do serve to decrease interspinal space and maximize bony support along the spinal column, it is possible that they represent an additional, independent evolution of a mechanism for spinal rigidity. Sauropods also exhibit neural spine modifications, such as bifurcating neural spines, which have been hypothesized to be an adaptation for increased vertebral column mobility [1]. Some sauropod specimens (most notably *Diplodocus longus* USNM 10865) possess spinal projections in the caudal series that are identical to those observed in large-bodied theropods [42]. In USNM 10865, the rugose projections are hypothesized to be osteological modifications associated with a highly pathologic caudal series; thus, it is thought that the spinal projections are a response from the need to horizontally maintain the caudal series [43]. Additionally, several titanosauriform specimens have been documented which retain biomineralized and non-biomineralized remnants of their vertebral ligaments [42,44]. The most extensive of these documented are reported by Cerda et al. (2015), in which nine biomineralized supraspinal ligaments are reported in titanosauriform sacra. These biomineralized ligaments are restricted to the apices of the sacral neural spines, and from histologic analysis, Cerda et al. (2015) conclude that biomineralization of the sacral vertebral ligaments occurs via metaplasia in direct response to ligament tension.

Our hypothesis predicts that metaplastic spinal projections should increase in size throughout ontogeny in large-bodied species. Limited data seem to support this prediction. For instance, immature *Tyrannosaurus rex* specimens exhibit neural spines with smaller, less developed projections compared with older individuals. This morphological difference could be an apomorphy [45], but it also agrees with the hypothesis outlined here. Within the genus *Ceratosaurus*, there are potentially three species: *C*. *nasicornis*, *C*. *dentisulcatus*, and *C*. *magnicornis*. There has been debate over the validity of these taxa, and the argument has been made that these “species” in fact represent ontogenetic morphotypes [46–48]. The smallest of the three, *C*. *nasicornis*, does not possess the metaplastic spinal projections, yet the larger *C*. *dentisulcatus* does. The development of metaplastic projections on neural spines in these species could be evidence that *C*. *nasicornis* represents the more immature animal, while *C*. *dentisulcatus* the more mature. Regardless, these data suggest that stress-induced metaplasia dorsoventrally stiffened the spinal column progressively over the lifespan of large-bodied species. In light of our findings, use of this feature as a character to infer phylogenetic relationships should be reconsidered. Future work integrating the results presented here with development, postural and locomotor biomechanics, and respiration, may help elucidate the paleobiology of non-avian theropods.

## Data Accessibility

All femoral length and rugosity presence/absence data, along with their cited references, are listed in the Table 1. The program used for our phylogenetic methods, BayesTraits can be downloaded at the following URL: http://www.evolution.rdg.ac.uk/BayesTraits.html. Histologic sections are housed at the Museum of the Rockies. Their accession numbers are specified in the Materials and Methods section.
